# Outcome after severe head injury: focal surgical lesions do not imply a better Glasgow Outcome Score than diffuse injuries at 3 months

**DOI:** 10.1186/1752-2897-3-5

**Published:** 2009-04-03

**Authors:** Paul Leach, Omar N Pathmanaban, Hiren C Patel, Julian Evans, Raphael Sacho, Richard Protheroe, Andrew T King

**Affiliations:** 1 Division of Neurosurgery, GMNC, Manchester Academic Health Sciences Centre, Salford Royal NHS Foundation Trust, University of Manchester, Manchester, UK; 2 Department of Neurointensive Care, GMNC, Manchester Academic Health Sciences Centre, Salford Royal NHS Foundation Trust, University of Manchester, Manchester, UK

## Abstract

**Background:**

Historically neurosurgeons have accepted head injured patients only in the presence of a mass lesion requiring surgical decompression. Underpinning this is an assumption that these patients have a better outcome than patients without a surgical lesion. This has meant that many patients without a surgical lesion have been managed locally in the referring hospital. However, there is now evidence that treatment of all head injured patients in a specialist centre leads to improved outcomes. Therefore, we have asked the question: does the presence of a surgical lesion imply better outcome from severe head injury?

**Results:**

We prospectively recorded the Glasgow Outcome score (GOS), at 3 months, of all the severely head injured patients treated at our institution over a two and a half year period. Of 116 patients admitted with an initial Glasgow Coma Score (GCS) of 8 or less, 58 had surgical lesions and 58 non-surgical head injuries. The two groups were well matched for presenting GCS and age. Overall our favourable outcome rate (GOS 4 and 5) at 3-months for the patients with a surgical lesion and for the non-surgical group were 47.3% and 46.6% respectively, with no significant difference between the two (P = 0.54).

**Conclusion:**

The assumption in the past has always been that patients presenting in coma from traumatic diffuse brain injury will do worse than those that have a mass lesion amenable to surgical decompression. Our series would suggest that this is not the case and all severely head injured patients should expect similar outcome when cared for in a neuroscience centre.

## Background

It is well accepted that prompt surgical decompression of traumatic intracranial haematomas leads to improved outcomes for head injured patients. [1-3] In keeping with this, recent prognostic models have identified non-evacuated intracranial haematomas as strong predictors of poor outcome in severe head injured patients.[4] As such, it is usual for patients with traumatic intracranial mass lesions to be transferred to a neurosciences centre for surgery and neurointensive care.

Conversely, due to lack of infrastructure, patients deemed to have non-surgical head injuries have often been managed outside of neuroscience centres. This approach to allocating limited resources is based on a long-held belief that individuals with focal surgical lesions will do better than those with diffuse injuries. Indeed, contemporary data predicts that individuals with extradural haematomas (EDH) will do better than patients with other types of severe closed head injury.[5] This is intuitive because an EDH compromises neural function by compression, but often has minimal associated underlying parenchymal injury; prompt surgical evacuation will thus lead to a good recovery.

However, biased resource allocation in favour of the surgical group is now controversial because it has been shown that the non-surgical group of severely head injured patients also fare better by treatment in a specialist centre.[6] Moreover, the prognostic advantage for EDH should not necessarily be extrapolated to other traumatic mass lesions such as acute subdural haematomas or contusions, where associated parenchymal injury is a usual feature.

Despite this, there remains a paucity of evidence in the literature on the expected outcome of severely head injured patients without a mass lesion as compared to those with a surgical lesion when both groups are treated in a specialist centre. Therefore, we have compared our outcomes for severely head injured patients with and without mass lesions.

## Results

### Study Cohorts

Over the study period 116 patients with an initial GCS of eight or less were admitted to Greater Manchester Neuroscience Centre. Ninety one patients were male and 25 female. The age range was 16–78 with the mean age being 36.2 years. Of these 116 patients, 58 had a mass lesion that required surgical evacuation and 58 had a non-surgical head injury. The age and presenting GCS of these two groups were compared using an independent sample 2-tailed t test and the Mann-Whitney U test respectively. The two cohorts were well matched for presenting GCS with no significant difference (P = 0.66). There was a statistically significant difference in age (P = 0.05), but not a biologically significant difference (see table 1).

**Table 1:** Mean ages and median initial presenting GCS of the surgical and non-surgical groups

	Surgical group	Non-surgical group	Statistical difference (P-Value)
Mean age (years)	39.1 (range 16–69)	33.3 (range 16–78)	0.05

Median initial GCS	4	5	0.66

### Glasgow Outcome Scores (GOS) at 3 months

Outcome data was collected for 115 patients as 1 patient in the surgical group was lost to follow up. The GOS at 3-months for the surgical group and for the non-surgical group are shown in tables 2 and 3 respectively. Our favourable outcome rate (GOS 4 and 5) at 3-months for the patients with a surgical lesion and for the non-surgical group were 47.3% and 46.6% respectively. To assess any difference in outcome between the surgical and non-surgical cohorts, we applied a Mann-Whitney U test to the dataset after dichotomising GOS into good (GOS 4–5) and poor (GOS 1–3) outcomes. There was no significant difference in outcome between the two groups (P = 0.54).

**Table 2:** Glasgow Outcome Scores at 3-months for the patients requiring surgical decompression treated in our unit over the study period

GOS	Number of patients	%
1	18	31.6

2	2	3.5

3	10	17.5

4	17	29.8

5	10	17.5

**Table 3:** Glasgow Outcomes Scores at 3-months for the patients with non-surgical severe head injury treated in our unit over the study period

GOS	Number of patients	%
1	22	37.9

2	1	1.7

3	8	13.8

4	19	32.8

5	8	13.8

## Discussion

We have demonstrated that a favourable outcome (GOS 4 or 5) for patients with non-surgical severe traumatic brain injury (46.6%) was not significantly different to the outcome for patients in the surgical cohort (47.3%) (P = 0.54). There are few studies that have explicitly and prospectively assessed outcome in patients with severe non-surgical head injury and therefore our results serve as a good reference point. In the Traumatic Coma Database study group [8] 85% of patients with diffuse injury had a poor outcome at hospital discharge which is significantly poorer than our results at three months. On the other hand, the overall outcome from other contemporary series might suggest that our outcomes fall short of the results from the 'best centres'. At the time of this study, our institution did not have a protocol driven head injury management scheme and in light of this, these results compare favourably with those reported from the pre-protocol era (see Table 4).

**Table 4:** Favourable outcomes (%) achieved in three different series of severely head injured patients prior to the institution of protocol driven therapy

Author	Epoch	Number of patients	Favourable outcome
Fakhry [9] et al	1991–1994	N = 219	43.3%

Patel [10] et al	1991–1993	N = 53	40.4%

Warme [11] et al	1980–1981	N = 49	32%

Historically neurosurgeons have accepted patients with major head injuries only in the presence of a mass lesion requiring surgical decompression. They have been accused of "cherry picking" and their good results may be attributed to this. It has recently been suggested that this approach is not appropriate as patients treated in a non-neurosurgical setting with a diffuse injury have a higher probability of death compared with those treated in a neurosurgical setting.[6] The role of neurosurgical care is further supported by the observation of higher mortality in patients operated on for an acute subdural haematoma who were transferred back to the ICU at the referring hospital due to lack of speciality beds.[12] What is more, Varelas et al found that the appointment of a neurointensivist to their neurosurgical ICU improved the outcomes of their head injured patients.[13]

Finally, there is a long held argument that aggressive, or specialist facilities lead to increased survival, but with a resultant increment in the number of patients left with severe disability or in a persistent vegetative state (PVS). The results here demonstrate in line with others that the PVS rate in patients with diffuse injury is very low.

## Conclusion

The assumption in the past has always been that patients presenting in coma from traumatic diffuse brain injury will do worse than those that have a mass lesion amenable to surgical decompression. Our series would suggest that this is not the case and all severely head injured patients should expect a similar outcome. Admission to a neuroscience centre impacts favourably on outcome and our data suggests the absence of a lesion requiring surgical decompression does not imply a poorer outcome. Therefore the authors are of the opinion that severely head injured people should be managed in a specialist centre regardless of the presence of a surgical lesion.

## Methods

Outcome data was collected at three months post injury for all patients with a head injury, presenting initially with a Glasgow Coma Score (GCS) of eight or less, admitted to our intensive care unit (ICU), over a two and a half year period (July 2003 – December 2005). The initial presenting GCS was taken as the first documented GCS from the time of injury. Data was recorded within 24 hours of admission to our unit as part of a routine auditing process. There was no specific admission policy to our unit for these patients over the study period. Patients were admitted to the unit at the discretion of the on-call Consultant Neurosurgeon. They were admitted for either decompression of a mass lesion or medical management of their intracranial pressure (ICP). Patients or relatives were contacted at 3 months via telephone and assessed by the lead author (PL) using the Glasgow Outcome Score (GOS).[7] Outcomes of patients requiring surgical decompression for a mass lesion were compared to the outcomes for the non-surgical group.

## Competing interests

The authors declare that they have no competing interests.

## Authors' contributions

PL and AK were responsible for the conception and design of the study. PL collected all data. All authors contributed to the analysis and interpretation of data which was initially performed by OP, PL and RS. OP and PL drafted the manuscript and all authors reviewed it critically for intellectual content and have given final approval of the version to be published.
